# Authors’ Reply to Unraveling the Place of Small Molecules in the Treatment of Fistulizing Crohn’s Disease—A Systematic Review and Network Meta-analysis

**DOI:** 10.1093/crocol/otad075

**Published:** 2023-12-13

**Authors:** Arshdeep Singh, Vandana Midha, Gursimran Singh Kochhar, Bo Shen, Ajit Sood

**Affiliations:** Department of Gastroenterology, Dayanand Medical College, Ludhiana, Punjab, India; Department of Medicine, Dayanand Medical College, Ludhiana, Punjab, India; Division of Gastroenterology, Hepatology, & Nutrition, Allegheny Health Network, Pittsburgh, PA, USA; Center for Interventional Inflammatory Bowel Disease, Columbia University Irving Medical Center, New York Presbyterian Hospital, New York, NY, USA; Department of Gastroenterology, Dayanand Medical College, Ludhiana, Punjab, India

Dear Editors

We read with great interest the letter to the editor by Attauabi et al. in response to our recently published review on the management of perianal fistulizing Crohn’s disease (FCD).^1^ We appreciate the opportunity to address the concerns raised by the reader and offer further clarification on the points raised.

The exploration of small molecules in the context of perianal FCD reflects a growing interest in developing targeted and effective pharmacotherapies to improve outcomes and quality of life for individuals affected by this challenging manifestation of Crohn’s disease (CD). The same was elaborated upon in our comprehensive review. The Janus kinase/signal transducers and activators of transcription (JAK/STAT) pathways have been reported to feature more prominently in patients with perianal FCD.^2^ We also discussed the Phase 2 DIVERGENCE study that evaluated filgotinib (oral JAK1 inhibitor) for the treatment of perianal FCD.^3^

Regarding the data from Phase 3 randomized controlled trials concerning upadacitinib in perianal fistulae and fissures, and its presentation as an abstract at the annual congress of European Crohn’s and Colitis Organization, 2023—beyond our literature search period—it is imperative to emphasize that both perianal and enterocutaneous fistula were included in the analysis.^4^ Notably, the remission and response rates for perianal fistula were not delineated separately. The findings in the reported network meta-analysis concerning the efficacy of upadacitinib in inducing remission, therefore, encompass the combined remission rates of both perianal and enterocutaneous fistulae. Additionally, the surface under the cumulative ranking (SUCRA) revealed maximum efficacy for infliximab in combination with ciprofloxacin, followed by infliximab monotherapy and adipose-derived stem cells, with upadacitinib ranking inferior to these therapies. Moreover, the authors themselves acknowledge that despite efficacy in the clinical trials, upadacitinib was not superior to placebo in the maintenance of fistula remission in the network meta-analysis. The fundamental tenet in the management of inflammatory bowel disease stipulates employing the same therapy for both inducing and maintaining remission. This principle dissuades the endorsement of upadacitinib for perianal FCD based on the results of the reported network meta-analysis.

The statement that infliximab, adipose-tissue-derived stem cells, or upadacitinib might be considered as potential options for achieving fistula remission warrants careful interpretation. The management of perianal FCD is intricate and continually evolving. According to the currently available evidence, anti-tumor necrosis factor agents (infliximab > adalimumab) remain the primary therapeutic choice for patients with perianal FCD. The universal recommendation of adipose-tissue-derived stem cells for all patients with perianal FCD is not feasible, given contraindications in individuals with active proctitis and requirement of specialized expertise to perform the procedure.

We acknowledge the promise of small molecules in the treatment of perianal FCD, as was also highlighted in the review article. However, at this moment, it would be premature to designate upadacitinib as the preferred drug. Further data from randomized controlled trials investigating oral small molecules in perianal FCD are required.

## Data Availability

Data sharing is not applicable to this article as no new data were created or analyzed in this study.

## References

[1] Singh A , Midha V, Kochhar GS, Shen B, Sood A. Management of perianal fistulizing Crohn’s disease. Inflamm Bowel Dis. 2023:izad195. doi:10.1093/ibd/izad195 37672347

[2] Kaur M , Panikkath D, Yan X, et al. Perianal Crohn’s disease is associated with distal colonic disease, stricturing disease behavior, IBD-associated serologies and genetic variation in the JAK-STAT pathway. Inflamm Bowel Dis. 2016;22(4):862–869. doi:10.1097/MIB.0000000000000705 26937622 PMC5220246

[3] Reinisch W , Colombel JF, D’Haens GR, et al. OP18 Efficacy and safety of filgotinib for the treatment of perianal fistulizing Crohn’s disease: results from the phase 2 DIVERGENCE 2 study. J Crohns Colitis. 2022;16(Suppl_1):i019–i021. doi:10.1093/ecco-jcc/jjab232.017 PMC11147792 38366672

[4] Colombel JF , Irving P, Rieder F, et al. P491 efficacy and safety of upadacitinib for the treatment of fistulas and fissures in patients with Crohn’s disease. J Crohns Colitis. 2023;17(Suppl_1):i620–i623. doi:10.1093/ecco-jcc/jjac190.0621

